# Autocrine Tnf signaling favors malignant cells in myelofibrosis in a Tnfr2-dependent fashion

**DOI:** 10.1038/s41375-018-0131-z

**Published:** 2018-04-18

**Authors:** William L. Heaton, Anna V. Senina, Anthony D. Pomicter, Mohamed E. Salama, Phillip M. Clair, Dongqing Yan, Russell N. Bell, Jeremy M. Gililland, Josef T. Prchal, Thomas O’Hare, Michael W. Deininger

**Affiliations:** 1 Huntsman Cancer Institute, University of Utah, Salt Lake City, UT USA; 2 ARUP Laboratories, University of Utah, Salt Lake City, UT USA; 3 Department of Orthopaedics, University of Utah, Salt Lake City, UT USA; 4 Division of Hematology and Hematologic Malignancies, University of Utah, Salt Lake City, UT USA

## Abstract

Tumor necrosis factor alpha (TNF) is increased in myelofibrosis (MF) and promotes survival of malignant over normal cells. The mechanisms altering TNF responsiveness in MF cells are unknown. We show that the proportion of marrow (BM) cells expressing TNF is increased in MF compared to controls, with the largest differential in primitive cells. Blockade of TNF receptor 2 (TNFR2), but not TNFR1, selectively inhibited colony formation by MF CD34^+^ and mouse JAK2^V617F^ progenitor cells. Microarray of mouse MPN revealed reduced expression of X-linked inhibitor of apoptosis (*Xiap*) and mitogen-activated protein kinase 8 (*Mapk8*) in JAK2^V617F^ relative to JAK2^WT^ cells, which were normalized by TNFR2 but not TNFR1 blockade. *XIAP* and *MAPK8* were also reduced in MF CD34^+^ cells compared to normal BM, and their ectopic expression induced apoptosis. Unlike *XIAP*, expression of cellular IAP (cIAP) protein was increased in MF CD34^+^ cells. Consistent with cIAP’s role in NF-κB activation, TNF-induced NF-κB activity was higher in MF vs. normal BM CD34^+^ cells. This suggests that JAK2^V617F^ reprograms TNF response toward survival by downregulating XIAP and MAPK8 through TNFR2. Our results reveal an unexpected pro-apoptotic role for XIAP in MF and identify TNFR2 as a key mediator of TNF-induced clonal expansion.

## Introduction

Myelofibrosis (MF) is a myeloproliferative neoplasm (MPN) characterized by bone marrow (BM) reticulin fibrosis, anemia, and splenomegaly due to extramedullary hematopoiesis. Constitutive activation of JAK/STAT signaling due to somatic mutations in Janus kinase 2 (JAK2), calreticulin (CALR) or the thrombopoietin receptor (MPL) is central to MF pathogenesis and endows neoplastic cells with a competitive advantage over their normal counterparts [1–6]. JAK2 inhibitors, such as ruxolitinib, reduce splenomegaly and symptom burden and may prolong survival in MF patients, but are not curative [7]. JAK/STAT signaling promotes generation of inflammatory cytokines such as tumor necrosis factor (TNF), which cause constitutional symptoms such as weight loss and fever [8]. We have shown that TNF stimulates myeloid colony formation by MF CD34^+^ cells but reduces colony formation by normal controls and that absence of TNF attenuates disease in mice with JAK2^V617F^-induced MPN [2].

The aging BM is characterized by inflammation, a bias toward myelomonocytic differentiation and somatic mutations in genes related to myeloid malignancies, including JAK2 [9–14]. This suggests that MPNs such as MF may arise through a process in which clones carrying JAK2 activating mutations are selected in the inflammatory environment, a concept supported by the steep increase of MPN incidence with age [15]. The fact that TNF mediates the proliferative advantage of MPN over normal cells suggests therapeutic utility for TNF antagonists. The TNF neutralizing agent etanercept improved symptoms in a pilot study of MF patients [16]. However, TNF inhibitors have not been developed as MF therapeutics, reflecting concerns about immunosuppression in an already immunocompromised patient population.

TNF signaling involves two distinct receptors, TNF receptor 1 (TNFR1) and TNF receptor 2 (TNFR2). TNFR1 expression is ubiquitous, while TNFR2 expression is largely confined to cells of hematopoietic origin. Only TNFR1 contains an intracellular death domain (DD) that is required for formation of a pro-apoptotic complex (Complex II) consisting of Fas-associated death domain (FADD), TNFR1-associated death domain (TRADD), receptor-interacting serine/threonine protein kinase 1 (RIPK1) and procaspase-8 [17]. Despite TNFR1’s ability to form this pro-apoptotic complex, the initial signaling event following TNF engagement is the formation of a membrane bound complex (Complex I) where TRADD associates with TNFR-associated factor 2 (TRAF2), cellular inhibitor of apoptosis (cIAP) and poly-ubiquitinated RIPK1 leading to nuclear factor-κB (NF-κB) activation. Subsequent formation of Complex II occurs after dissociation from TNFR1 and internalization to the cytoplasm [18]. Cell fate is dependent on factors that balance the NF-κB induction of inflammatory and survival signals with caspase-8-dependent apoptosis. Important factors are cIAP-mediated stabilization of Complex I and expression of the long isoform of cellular FLICE (FADD-like IL-1β-converting enzyme)-inhibitory protein (c-FLIP_L_), an inactive caspase-8 homolog that competes for binding to FADD, thereby maintaining Complex II in an inactive state [19, 20]. Since TNFR2 lacks the DD, it cannot associate with TRADD and FADD, however TNFR2 is able to bind TRAF2 and coordinate NF-κB signaling in association with cIAP [21].

The fact that TNF favors growth of MPN over normal cells suggests that JAK/STAT activation shifts TNF signaling outcomes to survival/proliferation, but it is unknown which differences in signaling between MPN and normal cells underlie the differential response to TNF. Here we show that blocking TNFR2 but not TNFR1 selectively inhibits MPN cells over normal controls and implicate X-linked inhibitor of apoptosis (XIAP), cIAP and mitogen-activated protein kinase 8 (MAPK8) as key mediators of differential responses to TNF.

## Methods

### Human samples

Peripheral blood (PB) and BM samples were collected according to an IRB-approved protocol (#45880), following informed consent. When isolating specific cell fractions, mononuclear cells (MNCs) were prepared with ficoll-paque (GE Healthcare, Uppsala, Sweden) and positive selection with microbeads (Miltenyi Biotec, Bergisch Gladbach, Germany) was performed on an autoMACS Pro Separator (Miltenyi). Details of MF patient information and normal BM donors are provided in Supplemental Tables 1–2.

### Intracellular TNF and TNF receptor (TNFR) staining

Leukocytes from MF (*n* = 5) or normal BM (*n* = 10) samples were treated with 10 μg/mL brefeldin A (Sigma-Aldrich) and 100 ng/mL lipopolysaccharide (LPS; Sigma-Aldrich), where indicated. Human antibody panels were used to identify stem and progenitor cells as described [22] and mature populations were identified using standard markers. Stem and progenitor cell populations in Balb/c mice (*n* = 8) were identified as reported [23] except for the omission of Sca-1 (low expression in Balb/c mice; complete list of markers and antibodies is provided in the Supplemental Tables 3–6). Cells were stained for surface markers prior to incubation in a paraformaldehyde/saponin buffer (BD Bioscience, Franklin Lakes, NJ), then stained with TNF antibody (BD Bioscience). Samples were analyzed using a FACSCanto cytometer (BD Bioscience) and analyzed with FlowJo analysis software (Treestar, Ashland, OR). Graphs show mean/s.e.m.

### Clonogenic assays

In murine samples, lineage depletion was performed with an autoMACS Pro Separator (Miltenyi Biotec), prior to sorting for DAPI^−^Kit^+^ positive cells with a FACSAria Cell Sorter (BD). Human CD34^+^ cells (MF-n = 4, CB-n = 3) or Lin^−^Kit^+^ mouse cells (*n* = 3) were treated in liquid culture with TNFR blocking antibodies (Supplemental Table 7) at 10 μg/mL for 72 h, then plated in MethoCult with continued antibody treatment. Liquid culture and colony assays were supplemented with SCF (human-50 ng/mL, murine-100 ng/mL) and IL-3 (human-10 ng/mL, murine-50 ng/mL). Inducible shRNA constructs were purchased from Cellecta (Mountain View, CA) in lentiviral vectors containing a GFP expression marker. GFP^+^ cells were sorted 3 days post infection (*n* = 3), and treated ±200 ng/mL doxycycline (Clontech Laboratories Inc., Mountain View, CA). Cells were maintained in liquid culture for 96 h, transferred to clonogenic assays as described above, with colonies scored after 10–14 days. Graphs show mean/s.e.m.

### Microarray analysis

BM cells were harvested from 3 to 4 mice for each experiment, cultured for 16 h ±TNFR1 or TNFR2 BA (10 μg/mL). After treatment, Lin^−^Kit^+^ cells were isolated as described above and further subdivided into GFP^+^ and GFP^−^ cells for a total of six distinct groups. Three independent experiments were performed and RNA was isolated using RNeasy Micro Kit (Qiagen, Germantown, MD). RNA was further purified using Clean & Concentrator columns (Zymo Research, Irvine, CA), cDNA was prepared with the Ovation Pico WTA System V2 (NuGEN, San Carlos, CA) and labeled with the Encore Biotin Module (NuGEN). The cDNA target samples were hybridized to an Affymetrix Mouse Expression 430 2.0 array (Thermo Fisher Scientific). Image processing was performed using Affymetrix Command Console (AGCC) v.3.1.1 software (Thermo Fisher Scientific) and expression analysis was performed using Affymetrix Expression Console build 1.4.1.46 (Thermo Fisher Scientific). Data have been uploaded to Gene Expression Omnibus (accession number GSE104792).

### Statistical analysis

Significance of individual comparisons was determined by Student’s *t*-test with paired comparisons for murine samples (JAK^V617F+^ and JAK2^V617F−^ cells from individual mice) and unpaired comparisons between human MF samples relative to normal controls (with test for variance). Two-tailed tests were used for all comparisons except for intracellular TNF expression (one-tailed) where the distribution was limited by zero values. A two-way ANOVA was used for comparison of the NFκB time-course experiment.

## Results

### TNF expression is increased in MPN cells from multiple hematopoietic cell compartments

**Fig. 1:**
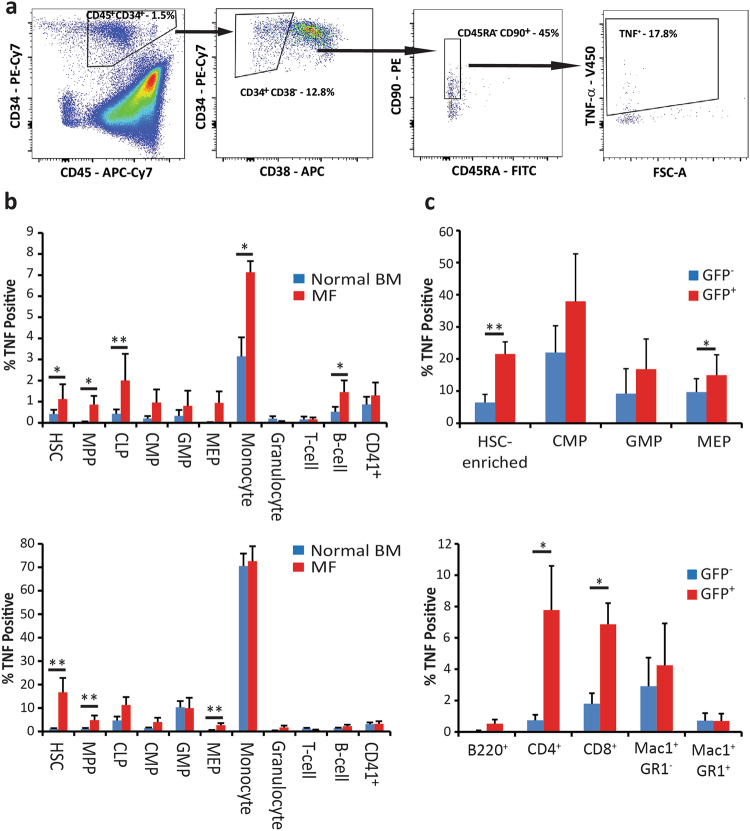
TNF expression is higher in HSCs from MF patients compared to normal BM and in cells from JAK2^V617F+^ compared to JAK2^V617F−^ mice. **a** Primary cells were immunophenotyped with surface markers and intracellular TNF staining was performed to identify expression in hematopoietic lineages, with HSCs defined as CD45^+^CD34^+^CD38^−^CD45RA^−^CD90^+^. **b** Leukocytes were treated *ex vivo* for 16 h with the protein transport inhibitor brefeldin A (10 μg/mL). The percentage of TNF^+^ cells was higher in MF patients (*n* = 5; PB = 4, BM = 1) compared to normal BM (*n* = 10) in HSCs, MPPs, CLPs, monocytes and B cells. **c** Addition of LPS (100 ng/mL) increased the differences in TNF expression between MF specimens (*n* = 5; PB = 4, BM = 1) and normal BM (*n* = 10) for HSCs, MPPs and MEPs while expression in monocytes was equivalent. **d**, **e** Similar experiments were performed with BM cells isolated from JAK2^V617F^ MPN mice (*n* = 8), including brefeldin A treatment. **d** TNF expression was significantly higher in JAK2^V617F+^ Lin^−^Kit^+^CD48^−^CD150^+^ HSC-enriched cells and Lin^−^Kit^+^CD34^−^FcγRII/RIII^Lo^ MEPs. **e** In the mature compartments, JAK2^V617F+^ T-cells (CD4^+^ and CD8^+^) expressed higher TNF than JAK2^V617F−^ T-cells, while expression in B cells (B220^+^) and myeloid cells (Mac-1^+^/GR-1^−^ and Mac-1^+^/GR-1^+^) was similar. *P < 0.05, **P < 0.005

To determine the cellular origin of TNF, we assessed expression by FACS in BM or blood of MF patients compared to normal controls (Fig. 1a). To increase intracellular TNF, allowing detection at the single-cell level, cells were treated with brefeldin A (10 μg/mL) [24, 25]. The percentage of TNF^+^ cells was increased in hematopoietic stem cells (HSCs), multipotent progenitor cells (MPPs), common lymphoid progenitor cells (CLPs), monocytes and mature B cells (Fig. 1b upper panel). To determine whether cell populations were differentially sensitive to inflammatory stimuli, cells were treated with LPS (100 pg/mL). TNF expression in monocytes from MF patients (72.5% TNF^+^) and normal individuals (70.5% TNF^+^) was comparable. In contrast, LPS-induced TNF expression in primitive populations was elevated in MF relative to normal controls, with the largest differential in HSCs (16.7% vs. 1.1% TNF^+^) and significant differences in MPPs and MEPs (Fig. 1b lower panel). Since the average age of the control population used in this study was significantly lower than the MF population (40.0 vs. 59.8 years old, *P* < 0.001), we also stratified normal controls based on age (<50 vs. >50 years old) and compared TNF expression. We found no difference in any of the populations, except for the LPS-stimulated granulocytes (*P* < 0.05), although the absolute expression was extremely low in this populations (<50 = 0.1% vs. >50 = 0.7% TNF^+^; Supplemental Figure 1). Analogous experiments were performed on BM from Balb/c mice with MPN induced by transplantation of donor cells transduced with JAK2^V617F^, where JAK2^V617F^ cells are identified by GFP expression [26, 27]. TNF expression was generally higher in JAK2^V617F^ cells, with the largest difference in the HSC-enriched population (21.5% vs. 6.4% TNF^+^ cells; Fig. 1c upper panel), irrespective of LPS stimulation. In mature populations, the largest difference in TNF expression between JAK2^V617F+^ and JAK2^V617F−^ cells was in T cells, while expression in the mature myeloid lineages (Mac1^+^ and/or GR1^+^) was comparable (Fig. 1c lower panel). These results indicate that the proportion of TNF expressing cells is consistently higher in MF vs. normal controls, particularly in less differentiated cell types. Furthermore, JAK2^V617F^ is sufficient to induce these differences in TNF expression.

### Treatment with pan-TNF inhibitors does not reduce disease burden in murine MPN

To test the effects of TNF neutralization *in vivo*, we used two TNF inhibitors in a retroviral mouse model of JAK2^V617F^-induced MPN. Etanercept is a soluble TNFR2 decoy receptor that binds human or mouse TNF, and has shown efficacy in mouse models of rheumatoid arthritis (RA) [28]. CNTO5048 is a modified version of the TNF-neutralizing antibody infliximab with an altered variable region that increases its affinity for mouse TNF [29]. Both agents are used to treat RA and other autoimmune disorders [30]. We confirmed the activity of each of these agents against mouse TNF in an assay of TNF-mediated cytotoxicity (L929 cells; Supplemental Figure 2a, b) [31]. In successive studies with etanercept and CNTO5048, mice with established MPN were treated for 8–12 weeks with dosing schedules reported to be effective in murine models of RA [28, 32]. Neutralization of soluble TNF in serum of mice was confirmed with the L929 cytotoxicity assay (Supplemental Figure 2c, d). No significant differences in white blood cell counts, hematocrit, GFP^+^ cells (Supplemental Figure 2e-j) or spleen weights (Supplemental Figure 2k,l) were observed between treatment groups. These results are consistent with the modest effects observed with etanercept in MF that showed hematologic improvement in 20% of patients [16]. Since the TNF neutralizing agents failed to inhibit disease, we hypothesized that differential signaling from TNFR1 and/or TNFR2 underlies the competitive advantage of MPN cells.

### TNFR2 but not TNFR1 selectively suppresses myeloid colony formation by MPN progenitor cells

**Fig. 2:**
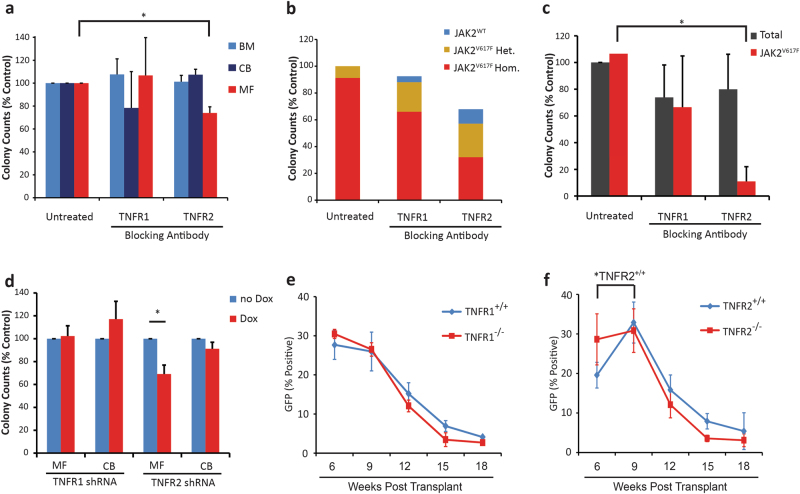
Blocking TNFR2 selectively inhibits colony formation by MF vs. normal BM CD34^+^ cells and mouse JAK2^V617F+^ compared to JAK2^V617F−^ progenitor cells. **a** Human CD34^+^ cells from MF blood (*n* = 4), normal BM (*n* = 3) or CB (*n* = 3) were treated with TNFR BAs at 10 μg/mL for 72 h in liquid culture, then plated in clonogenic assays with continued treatment. TNFR2 inhibition reduced colony numbers in MF samples without affecting normal BM or CB control samples. **b** In MF samples, the JAK2 genotype was analyzed for each colony. Of the four samples tested, three were found to have 100% JAK2^V617F^ allele burden in all conditions tested, while one sample with a mixed JAK2 genotype had a reduction in JAK2^V617F+^ colonies and increase in JAK2^WT^ colonies with TNFR2 inhibition and to a lesser degree with TNFR1 inhibition. **c** Lin^−^Kit^+^ BM cells from MPN mice (*n* = 3) were treated in liquid culture with TNFR BAs at 10 μg/mL for 72 h, then plated in clonogenic assays with continued treatment. Colonies were enumerated after 10 days and isolated colonies genotyped for JAK2 status by analyzing GFP expression. TNFR2 inhibition selectively reduced colony formation of JAK2^V617F+^ cells without affecting total colony numbers, while TNFR1 inhibition had no effect. **d** MF (*n* = 4) and CB (*n* = 4) CD34^+^ cells were infected with doxycycline-inducible TNFR shRNAs, in liquid culture ±200 ng/mL doxycycline for 96 h and then plated in clonogenic assays with continued treatment. Induction of the TNFR2 shRNAs reduced colony formation in MF samples without affecting normal controls, while induction with a TNFR1 shRNA had no effect. **e**, **f** BM cells from 5-FU-treated CD45.1 and **e** TNFR1^−/−^ or **f** TNFR2^−/−^ CD45.2 mice were infected with MSCV-IRES-JAK2^V617F^-GFP retrovirus. Equal numbers of wild type and null GFP^+^ cells were injected into lethally irradiated TNFR^+/+^ recipients (*n* = 8 per group). TNFR2^+/+^ GFP^+^ cells increased over TNFR2^−/−^ GFP^+^ cells, while TNFR1^+/+^ GFP^+^ cells remained at the same level as TNFR1^−/−^ cells. However, GFP^+^ engraftment was not maintained. *P < 0.05

To identify the mechanistic basis for the differential effects of TNF on MF vs. normal cells, we quantified TNF receptor expression in primitive (Supplemental Figure 3a) and mature (Supplemental Figure 3b) hematopoietic cells from human MF vs. normal BM and JAK2^V617F+^ vs. JAK2^V617F−^ cells from MPN mice (Supplemental Figure 3c). No significant differences were observed, suggesting that the differential effects of TNF on MPN vs. normal cells are due to modulation of downstream signaling events. To determine the contribution of TNFR1 and TNFR2-mediated effects on MPN clonal dominance, we used receptor blocking antibodies (BAs) to inhibit each receptor. The specificity of all BAs was confirmed using ELISA and L929 cytotoxicity assays (Supplemental Figure 4a-d). CD34^+^ cells from MF samples, normal BM or cord blood (CB) were cultured for 72 h ±TNFR1 or TNFR2 BAs (10 μg/mL), then transferred to semisolid medium with continued treatment. Blocking TNFR1 did not significantly change granulocyte-macrophage (GM) colony numbers for MF, BM, or CB samples, while TNFR2 block consistently reduced MF colonies by 25–30%, with no effect on BM or CB (Fig. 2a; colony counts provided in Supplemental Figure 5a-c). Single colonies were genotyped for JAK2 by BsaXI digestion (Supplemental Figure 6a). The vast majority of samples were homogenous for JAK2^V617F^ colonies. In one MF patient sample (from a patient with a JAK2^V617F^ allele burden of ~85%) with a mixture of JAK2^V617F^ and JAK2^WT^ colonies, blocking TNFR2 selected for JAK2^WT^ over JAK2^V617F^ colonies (Fig. 2b). We next tested the effects of TNFR BAs on Lin^−^Kit^+^ BM cells from mice with JAK2^V617F^ MPN, using the same experimental design. After enumeration, 20–25 single GM colonies were genotyped using a FACS-based assay measuring GFP (Supplemental Figure 6b, c). Both BAs mildly reduced total colony numbers. While the TNFR1 BA reduced JAK2^V617F+^ and JAK2^V617F−^ colonies to a similar degree, the TNFR2 blocking antibody selectively reduced JAK2^V617F+^ colonies (Fig. 2c). For validation, human MF and CB CD34^+^ cells were infected with doxycycline-inducible shRNAs targeting TNFR1 or TNFR2 (Supplemental Table 8). Effective TNFR1/2 knockdown was confirmed in SET-2 cells (Supplemental Figure 7a, b). Cells were cultured in cytokine containing media (SCF and IL-3, no exogenous TNF) ±doxycycline prior to plating in colony assays with continued treatment. Knockdown of TNFR2 reduced colony formation by MF cells to a similar degree as seen with the TNFR2 BA (~ 30%) with no effect on CB-derived colonies, while knockdown of TNFR1 had no effect (Fig. 2d). Altogether these results suggest that the differential effects of TNF on MPN vs. normal progenitor cells are mediated by TNFR2 and that selective inhibition of TNFR2 may reverse the clonal dominance of MPN cells. To test this, we performed a competitive repopulation assay with BM cells from TNFR^+/+^ (CD45.1_C57BL/6) and TNFR2^−/−^ (CD45.2_C57BL/6) mice. Analogous experiments were done using TNFR^+/+^ vs. TNFR1^−/−^ donors. Engraftment of GFP^+^ cells was monitored over time for TNFR^+/+^ and TNFR2^−/−^ compartments with CD45.1 and CD45.2 specific antibodies (Supplemental Table 9). While the proportion of TNFR1^+/+^ and TNFR1^−/−^ cells within the GFP^+^ population remained constant (Fig. 2e), JAK2^V617F^ expressing TNFR2^+/+^ outcompeted TNFR2^-/-^ cells (Fig. 2f). However, engraftment of GFP^+^ cells was transient and eventually JAK2^V617F+^ cells were lost irrespective of genotype, precluding further analysis.

### TNFR1 or TNFR2 inhibition partially reverses gene expression differences between JAK2^V617F+^ and JAK2^V617F−^ cells

**Fig. 3:**
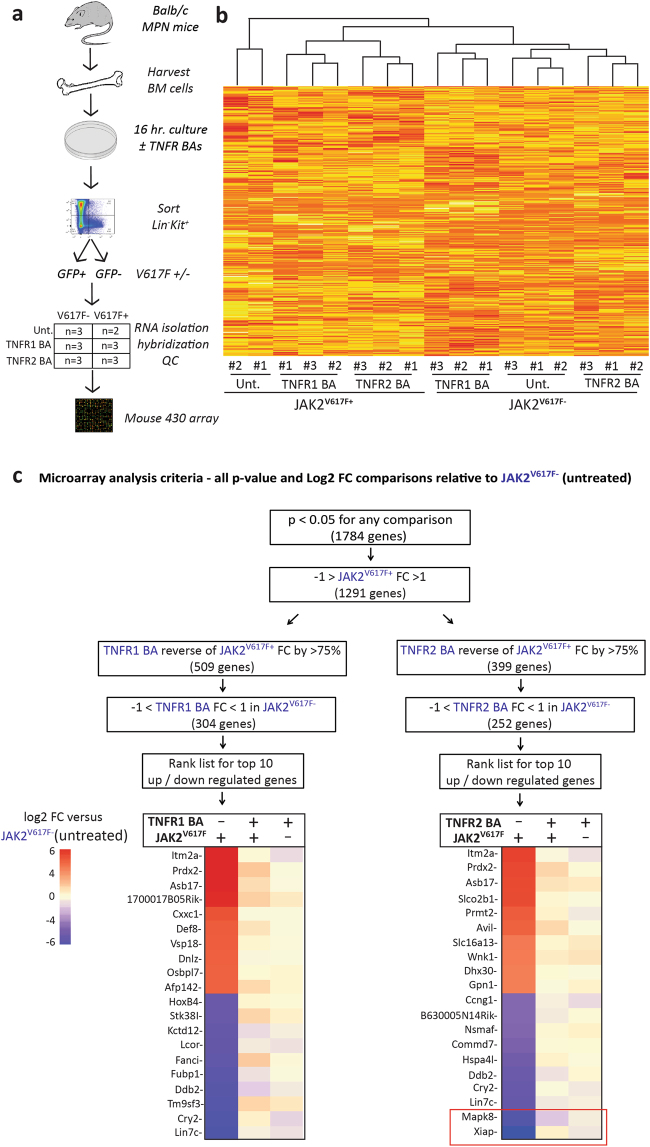
Expression of Xiap and Mapk8 is downregulated in JAK2^V617F+^ relative to JAK2^V617F−^ cells, but this differential is abolished by TNFR2 inhibition. **a** BM cells from mice with JAK2^V617F^-induced MPN was cultured for 16 h  ±TNFR BAs (10 μg/mL). Cells were then sorted for Lin^−^Kit^+^ expression, then subdivided based on GFP, resulting in six groups. Three independent experiments were performed. RNA was extracted for all 18 samples and subjected to microarray analysis using Affymetrix mouse 430 2.0 arrays. One sample, Untreated-JAK2^V617F+^ replicate #3 failed quality standards and was removed from further analysis. **b** Unsupervised clustering of the remaining 17 samples grouped all samples according to genotype (JAK2^V617F^) and treatment. **c** Fold change (FC) and *P*-values were generated for each condition relative to the JAK2^V617F−^ (Untreated) group. Sequential filters were applied to identify genes whose expression is dysregulated in JAK2^V617F+^ cells and restored with TNFR BA treatment. The set was limited to genes that had a *P*-value of < 0.05 when compared between any two treatment groups. Then genes were limited to those that had a FC > |1| in JAK2^V617F+^ relative to JAK2^V617F−^ cells. Genes were then subdivided into those whose expression was reversed with either TNFR1 or TNFR2 BA in JAK2^V617F+^ toward JAK2^V617F−^ by ≥75%. To further identify those that were selectively regulated in JAK2^V617F+^ cells, those with a FC > |1| in JAK2^V617F−^ cells were eliminated. For each of these gene sets we ranked the top 10 up- or downregulated genes. Xiap and Mapk8 were the two top differentially expressed genes between JAK2^V617F+^ and JAK2^V617F−^ whose expression was normalized with TNFR2 BA treatment but not TNFR1 BA treatment.

To identify the differences in TNF-induced signaling between JAK2^V617F+^ and JAK2^V617F−^ cells, we cultured BM cells from Balb/c mice with established JAK2^V617F^-induced MPN in media containing SCF and IL-3, ±murine TNFR1 or TNFR2 BA in three independent experiments (Fig. 3a). Lin^−^Kit^+^ cells were sorted into GFP^+^ and GFP^−^ subsets and subjected to gene expression profiling. All but one sample (untreated-JAK2^V617F+^ replicate #3, which was excluded) passed quality thresholds. Unsupervised hierarchical clustering grouped samples according to genotype (JAK2^V617F+^ vs. JAK2^V617F−^) and treatment (Fig. 3b). We hypothesized that genes critical to TNFR2′s distinct effects would be differentially expressed in JAK2^V617F+^ vs. JAK2^V617F−^ cells and that blocking TNFR2 should reverse this difference. In addition, we considered genes regulated by TNFR2 in JAK2^V617F+^ cells with minimal effects in JAK2^V617F−^ cells as the highest priority and applied sequential filters to identify genes meeting these criteria for TNFR2 and TNFR1, respectively (Fig. 3c). Evaluation of the top 10 up- and downregulated genes, based on fold-change between JAK2^V617F+^ vs. JAK2^V617F−^ cells, showed that expression of the genes with the highest degree of upregulation (*Itm2a*; *Prdx2*; *Asb17*) was reversed by either TNFR1 or TNFR2 block, while the two genes with the highest downregulation, *Xiap* and *Mapk8* (*Jnk1*), were selectively reversed by TNFR2 but not TNFR1 block (Fig. 3c). We therefore focused on *Xiap* and *Mapk8*, as genes potentially mediating the differential effects of TNF signaling in JAK2^V617F+^ vs. JAK2^V617F−^ cells. While it is conceivable that other differentially expressed genes such as *Prdx2* are also disease relevant, they were not investigated in the current study since they were not differentially regulated by TNFR2 over TNFR1.

**Fig. 4:**
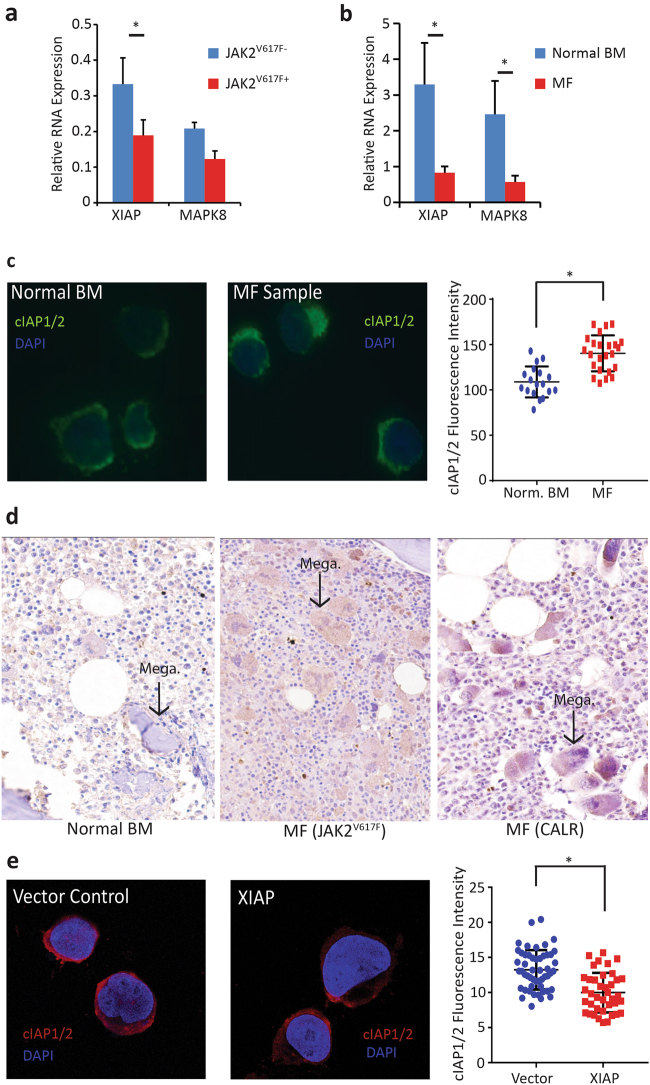
XIAP and MAPK8 mRNA expression is downregulated in mouse JAK2^V617F+^ cells and MF cells. **a** *Xiap* and *Mapk8* mRNA expression was measured by qPCR, using glyceraldehyde 3-phosphate dehydrogenase (*Gapdh*) as a control gene, in Lin^-^Kit^+^ cells from mice with JAK2^V617F^-induced MPN (*n* = 3). *Xiap* expression was significantly lower and *Mapk8* expression trended lower (*p* = 0.087) in JAK2^V617F^ cells. **b** *XIAP* and *MAPK8* expression was measured by qPCR, using β-glucuronidase (*GUS*) as a control gene, in MF (*n* = 5) and normal BM CD34^+^ cells (*n* = 3). Expression of both genes was significantly lower in MF. **c** Immunofluorescent images of CD34^+^ cells stained with a cIAP1/2 antibody. Fluorescence intensity was higher in MF (*n* = 4) cells compared to normal BM (*n* = 3). **d** Core BM biopsy sections from normal controls (*n* = 5), JAK2^V617F^ positive MF (*n* = 4) or CALR positive MF (*n* = 4) samples were stained with a cIAP1/2 antibody to evaluate expression. cIAP staining was stronger in both JAK2^V617F^ and CALR positive MF samples compared to the normal controls, particularly in the megakaryocytes (indicated by the black arrows). **e** Immunofluorescent images of MF cells (*n* = 3) with ectopic XIAP expression showed reduced cIAP1/2 fluorescence intensity relative to vector control. *P < 0.05

To validate the microarray results, we analyzed by qPCR *Xiap* and *Mapk8* expression in Lin^-^Kit^+^ cells from an independent group of MPN mice (*n* = 3) and in MF patients and normal BM controls (primer sequences are provided in Supplemental Table 10). Expression of *Xiap* and *Mapk8* was consistently lower in JAK2^V617F+^ compared to JAK2^V617F^^−^ murine cells and in MF cells compared to normal BM (Fig. 4a, b). MAPK8 has been implicated as a necessary component of TNF-mediated apoptosis [33], predicting that reduced expression would have an anti-apoptotic effect. Although XIAP inhibits caspase activity, low expression of XIAP is permissive for stabilization of the related family member cIAP. IAPs are known to regulate each other’s expression through their E3 ubiquitin ligase activity [34] and *Xiap* null mice show markedly increased cIAP protein [35]. Since cIAP is required for TNF-dependent NF-κB signaling [36], we hypothesized that the growth advantage of MPN cells exposed to TNF could be mediated through an increase in cIAP protein levels. We evaluated cIAP expression in CD34^+^ cells from MF samples and normal BM using immunofluorescence and found that cIAP expression was higher in MF samples relative to normal BM (Fig. 4c). Concordantly, cIAP staining was increased in BM cores from MF patients relative to normal controls (Fig. 4d). To validate the inverse relationship between XIAP and cIAP, we overexpressed XIAP in CD34^+^ cells from MF patient samples and measured cIAP expression. Overexpression of XIAP reduced cIAP levels relative to vector control (Fig. 4e).

### Blocking cIAP inhibits survival, while ectopic expression of XIAP or MAPK8 induces apoptosis in primitive MF cells

**Fig. 5:**
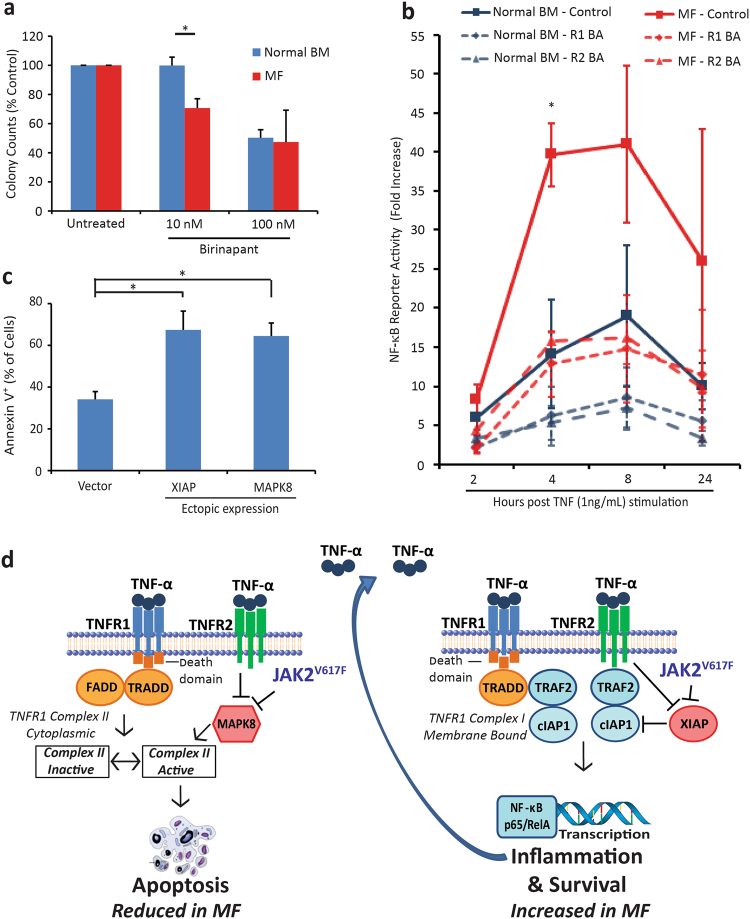
JAK2^V617F^ and TNFR2 cooperate to increase NF-κB signaling and reduce apoptosis in MF cells. **a** Human CD34^+^ cells isolated from MF patient (*n* = 3) or normal BM (*n* = 3) were treated with birinapant at 10 or 100 nM for 72 h in liquid culture and then plated in clonogenic assays with continued treatment. Colony inhibition was significantly different for normal vs. MF samples at 10 nM, while 100 nM birinapant inhibited both. **b** Human CD34^+^ cells isolated from MF patient (*n* = 3) or normal BM (*n* = 3) were infected with an NF-κB luciferase reporter construct 72 h prior to evaluation. Cells were treated with TNFR1 or TNFR2 BA (10 μg/mL) prior to stimulation with TNF (1 ng/mL). The fold increase in reporter activity was significantly higher in MF cells at 4 h post stimulation and over the complete time course (*P* = 0.03). Both TNFR1 and TNFR2 BAs reduced TNF stimulated NF-κB activity in MF and normal BM cells. **c** Annexin V was measured in MF CD34^+^ cells (*n* = 4) 72 h after infection with XIAP, MAPK8 or vector control expression constructs. Ectopic expression of XIAP or MAPK8 significantly increased Annexin V staining relative to vector control. **d** Downregulation of MAPK8 by JAK2^V617F^ and TNFR2 inhibits apoptotic signaling through TNFR1 by preventing MAPK8 from promoting the active form of TNFR1 Complex II. Downregulation of XIAP by JAK2^V617F^ and TNFR2 is associated with increased cIAP protein levels. Either TNFR1 (with TRADD) or TNFR2 can form a signaling complex through association with TRAF2 and cIAP to activate NF-κB transcription of pro-survival and inflammation-associated genes. These combined effects favor survival of JAK2^V617F^ cells. *P < 0.05, **P < 0.005

To test whether cIAP is functionally important, we used birinapant at a concentration that selectively inhibits cIAP over XIAP (10 nM) [37, 38]. CD34^+^ MF and normal BM cells were treated in liquid culture for 72 h then transferred to semi-solid media. Colony formation was significantly reduced in MF samples relative to normal BM, suggesting that cIAPs favor survival of MPN over normal cells (Fig. 5a). In contrast, 100 nM birinapant inhibited colony growth of MF and normal BM to a similar degree. Since cIAPs mediate TNF-induced NF-κB activation [36], we next assessed NF-κB signaling. MF (*n* = 3) or normal BM (*n* = 3) CD34^+^ cells were infected with an NF-κB reporter construct, pretreated ±TNFR1 or TNFR2 BA (10 μg/mL) followed by addition of TNF (1 ng/mL). NF-κB activity was maximal at 8 h post TNF stimulation with a 41-fold increase in MF cells and a 19-fold increase in normal BM. NF-κB activity was significantly higher in MF cells over the entire time course (*P* = 0.03). Treatment with either the TNFR1 or TNFR2 BA reduced NF-κB activity in MF and normal BM cells. Notably, either blocking antibody reduced NF-κB activity in MF cells to levels seen in the normal BM cells (Fig. 5b). We next ectopically expressed XIAP and/or MAPK8 in CD34^+^ cells from MF patient samples (*n* = 4) and measured annexin V after 72 h. Expression of MAPK8 and XIAP significantly increased annexin V^+^ cells over vector control, suggesting that their downregulation is critical for MPN cells to avoid TNF-induced apoptosis (Fig. 5c).

## Discussion

Chronic low-level inflammation is a feature of the aging hematopoietic system [9, 14, 39–43]. The BM concentrations of key inflammatory cytokines such as TNF and IFNγ increase with age and induce a myelomonocytic differentiation bias [9]. Concentrations of inflammatory cytokines are elevated in the plasma of most MPN patients. The highest levels are found in MF and correlate with symptom burden and shorter survival [44]. We have previously shown that TNF mediates the clonal dominance of JAK2^V617F^ over JAK2^WT^ cells, implicating TNF as a disease driver [2]. Although many inflammatory cytokines are elevated in MF, these data suggest a central role for TNF in MPN.

We show that TNF expression is higher in most cellular subsets of MF compared to controls under basal conditions, with differences enhanced by LPS in most hematopoietic progenitor cells. This is consistent with recent data showing that different hematopoietic cells have distinct cytokine production profiles including TNF [8]. These results suggest that MPN HSCs express TNF to inhibit the growth of normal HSCs competing for the same niches, especially under systemic inflammatory stress (LPS stimulation). These studies included samples from MF patients with either JAK2^V617F^ or CALR mutations, without appreciable differences between genotypes (patient profiles are provided in Supplemental Table 9).

TNF scavengers, such as etanercept and infliximab are associated with increased risk for opportunistic infections, giving rise to concerns about their use in immunocompromised patients, such as MF [45]. Data on anti-TNF therapeutics in MF are limited to one small study and anecdotal cases [16, 46]. Consistent with their modest effects in MF patients, etanercept and infliximab failed to reduce MPN disease in mice with JAK2^V617F^ MPN (Supplemental Figure 2). These results are at odds with the attenuation of MPN in a previous study using TNF deficient mice [2] and suggest that additional, unintended effects, of pan-TNF antagonists may neutralize differences in TNF responses between MF and normal cells [2, 47]. Several studies have implicated TNF as a negative regulator of HSCs [48–50]. Conflicting data were reported regarding the requirement for TNFR1 to mediate the suppressive TNF effects on HSCs cultured *ex vivo* [49, 50]. In competitive repopulation experiments, both TNFR1^−/−^ and TNFR2^−/−^ HSCs were shown to outcompete TNFR^WT^ cells to a similar degree, with a more pronounced advantage for double null cells [51]. To understand how JAK2^V617F^ reprograms TNF signaling in hematopoietic progenitors from suppression to stimulation, we asked whether this involves altered responses through TNFR1 and/or TNFR2. Our results support a model in which signaling through TNFR2, but not TNFR1, mediates the differential effects of TNF on myeloid colony formation of JAK2^V617F^ vs. JAK2^WT^ progenitor cells. Although the MF-specific reduction in colony formation with TNFR2 BA or knockdown was relatively modest (~30%; Fig. 2a, b, d), this difference may be sufficient to promote clonal dominance during the slow evolution of MPN. For unknown reasons, TNFR2 block reduced the JAK2^V617F+^ colonies much more profoundly in assays of mouse progenitor cells (by 89%, Fig. 2c). It is conceivable that the co-culture of JAK2^V617F^ and JAK2^WT^ cells is necessary to potentiate the differential effect and thus the difference was less dramatic in the human samples where MF and normal cells were cultured individually, in line with the observation that cytokine expression is altered both in mutant and normal cells isolated from MPN mice [8]. It is also possible that the over-expression of JAK2^V617F^ induced by the transduction/transplantation model may have amplified the effect in the mouse cells relative to the human samples. We selected this model because plasma TNF concentrations are elevated over controls to a similar degree as in MF (~10–15-fold) [26, 47].

The fact that TNFR1 and TNFR2 expression is comparable between MPN and normal BM cells excludes JAK/STAT regulation of TNFR1/2 expression as the cause of differential TNF responses, instead implicating differences in TNF signaling cascades. We found that expression of *Xiap* and *Mapk8*, two regulators of TNF signaling, is reduced in JAK2^V617F+^ mouse progenitor cells and that blocking TNFR2 restores their expression to the levels in JAK2^WT^ cells (Fig. 3a–c). Consistent with the mouse experiments, expression of *XIAP* and *MAPK8* was lower in human MF vs. normal BM CD34^+^ cells (Fig. 4b). Reduced expression of *Xiap* in murine JAK2^V617F+^ cells could be considered counterintuitive as XIAP inhibits caspases and hence apoptosis [52]. However, there is evidence for mutual regulation between XIAP and cIAP. Thus cIAP expression is increased in *Xiap* null mice, and this has been linked to the ability of XIAP to promote cIAP ubiquitination and proteasomal degradation, and vice versa [34, 35]. Indeed, cIAP expression was higher in MF compared to normal CD34^+^ cells and BM biopsies. Attempts to assess XIAP protein levels in hematopoietic cells were unsuccessful, but consistent with above predictions, ectopic expression of XIAP-induced apoptosis in MF CD34^+^ cells (Fig. 5c). Similarly, in many cancer cell lines, IAP proteins inhibit apoptosis induced by TNF [53, 54]. Downregulation of cIAPs or treatment with a cIAP inhibitor allows TNF-dependent apoptosis to proceed. This is reminiscent of our findings in MF cells, which are more sensitive than control to inhibition with the IAP inhibitor birinapant, at concentrations that block cIAP, but not XIAP; while the differential is lost at higher concentrations that block both (Fig. 5a). cIAP promotes activation of NF-kB and, as predicted, NF-kB reporter activity was shown to be higher in MF cells relative to normal BM cells (Fig. 5b) as reported by others [55]. This is consistent with data for the cIAP inhibitor LCL161, which is demonstrating activity in early clinical testing for the treatment of MF [56]. A second entry point into TNFR signaling involves reduction of MAPK8 expression to inhibit apoptotic signaling through TNFR1 Complex II. As MAPK8 promotes degradation of c-FLIP_L_ [20], reduced MAPK8 should stabilize the c-FLIP_L_ heterodimer with procaspase-8, favoring the inactive form of TNFR1 Complex II (Fig. 5d).

Altogether our findings are consistent with a model, in which MF cells downregulate XIAP and MAPK8 through a TNF/TNFR2-dependent autocrine loop to escape an apoptotic response and enhance NF-κB signaling (Fig. 5d). Since NF-κB is also a key mediator of inflammatory cytokine expression [57], this creates a positive feedback loop where TNF functions as a master regulator of inflammatory cytokine production in MPN cells. Studies to determine exactly how JAK2^V617F^ and other MF-associated mutations modulate TNFR2 signaling to limit the expression of XIAP and MAPK8 compared to their normal competitors are in progress. Our work supports the further clinical development of cIAP inhibitors and implicates selective TNFR2 inhibitors as potential therapeutics in the treatment of MF.

## Electronic supplementary material

Supplemental Material
