# Supplementary material for: Autocrine Tnf signaling favors malignant cells in myelofibrosis in a Tnfr2-dependent fashion
Source: Leukemia. 2018 Apr 18;32(11):2399–411. doi: 10.1038/s41375-018-0131-z (PMC6224399; doi:10.1038/s41375-018-0131-z)
Supplement: Supplementary file 1 — Supplemental Material [file 41375_2018_131_MOESM1_ESM.pdf]

Supplemental Figures

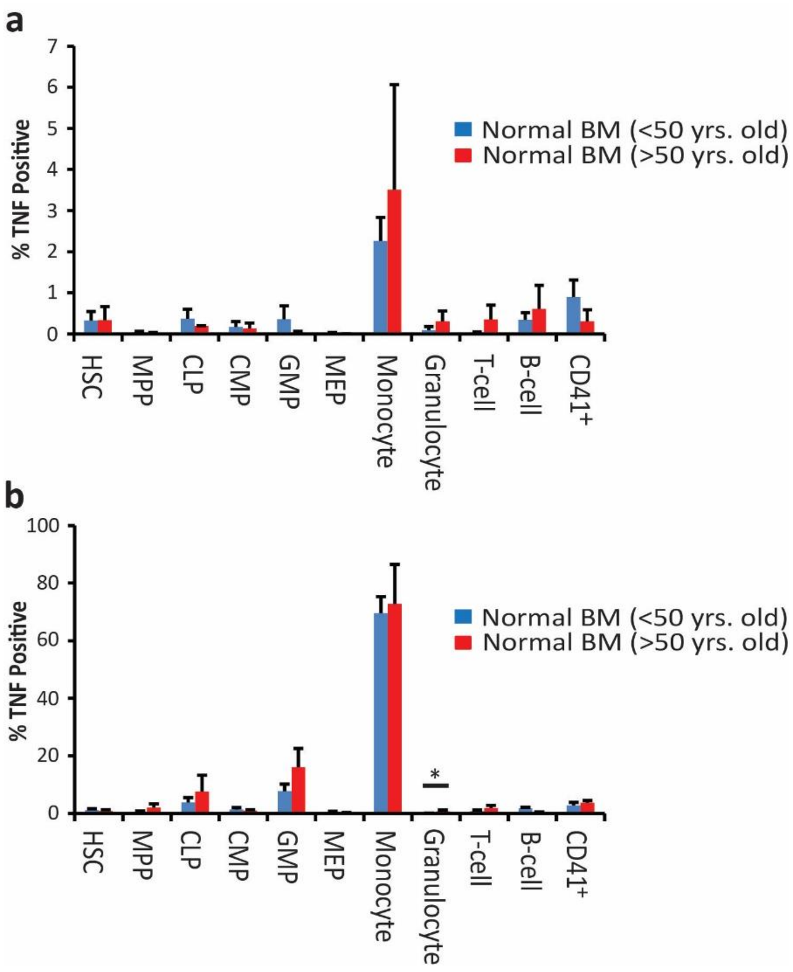

Supplemental Figure 1. TNF expression is equivalent in normal control groups stratified by age. (a,b) Data from normal BM controls (Fig. 1b,c) were divided into 2 groups based on the age of the donor (<50 or >50 years old). (a) There was no difference in TNF expression between the age groups for cells treated with brefeldin A only. (B) There was no difference in any of the groups treated with brefeldin A and LPS, except for the granulocyte population, although the absolute magnitude of TNF expressing cells in this group was extremely low (<50 = 0.1% vs. >50 = 0.7% TNF^{+}).*$p<0.05$

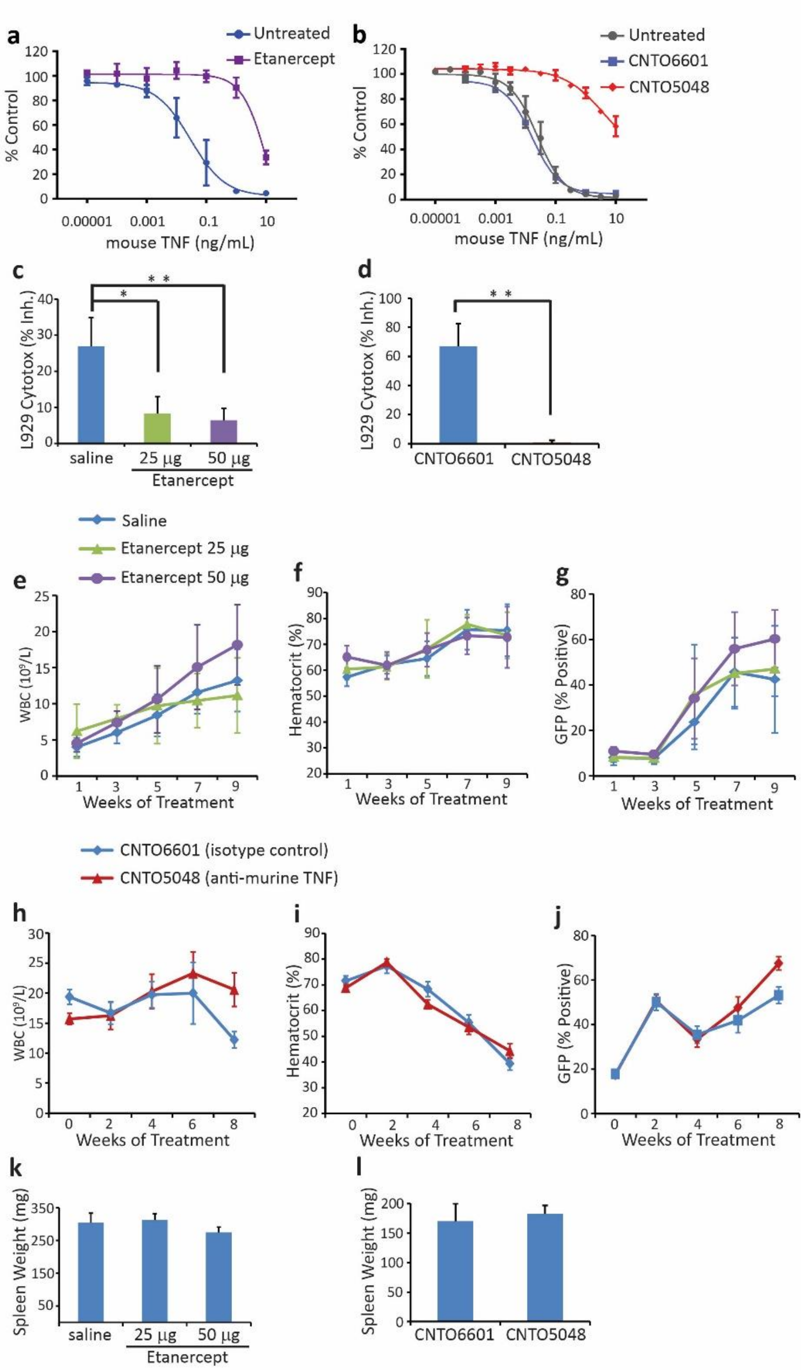

Supplemental Figure 2. Non-selective anti-TNF therapy does not reduce disease burden in MPN mice. (a) Etanercept (1 µg/ml) protected cells from the cytotoxicity of mouse TNF in L929 cells. (b) Mouse TNF cytotoxicity was inhibited by the murine version of infliximab (CNTO5048, 1 µg/mL) but not by the isotype control (CNTO6601, 1 µg/mL). (c,d) At the end of the study neutralization of TNF in plasma of mice treated with (c) etanercept or (d) CNTO5048 was confirmed by testing sterilized plasma samples from mice in the L929 cell TNF cytotoxicity assay. (e-g) Mice were treated every other day with saline (n=9), 25 µg etanercept (n=5) or 50 µg etanercept (n=12) by subcutaneous injection. Blood counts were analyzed every other week following sub-mandibular collection and the proportion of GFP^+ cells was

quantified as a percentage of total white blood cells (WBCs). There was no significant difference between the treatment groups with respect to (e) WBC counts, (f) hematocrit or (g) JAK2^{V617F} allele burden as measured by GFP expression. (h-j) Mice were treated with a monoclonal antibody selective for mouse TNF (CNTO5048; n=11) or isotype control (CNTO6601; n=11) at 8 mg/kg with intraperitoneal dosing twice a week. There was no significant difference between the treatment groups with respect to (h) WBC counts, (i) hematocrit or (j) JAK2^{V617F} allele burden. There was no significant difference in spleen weight between the treatment groups in the (k) etanercept study or (l) CNTO5048 study. *p<0.05, **p<0.005

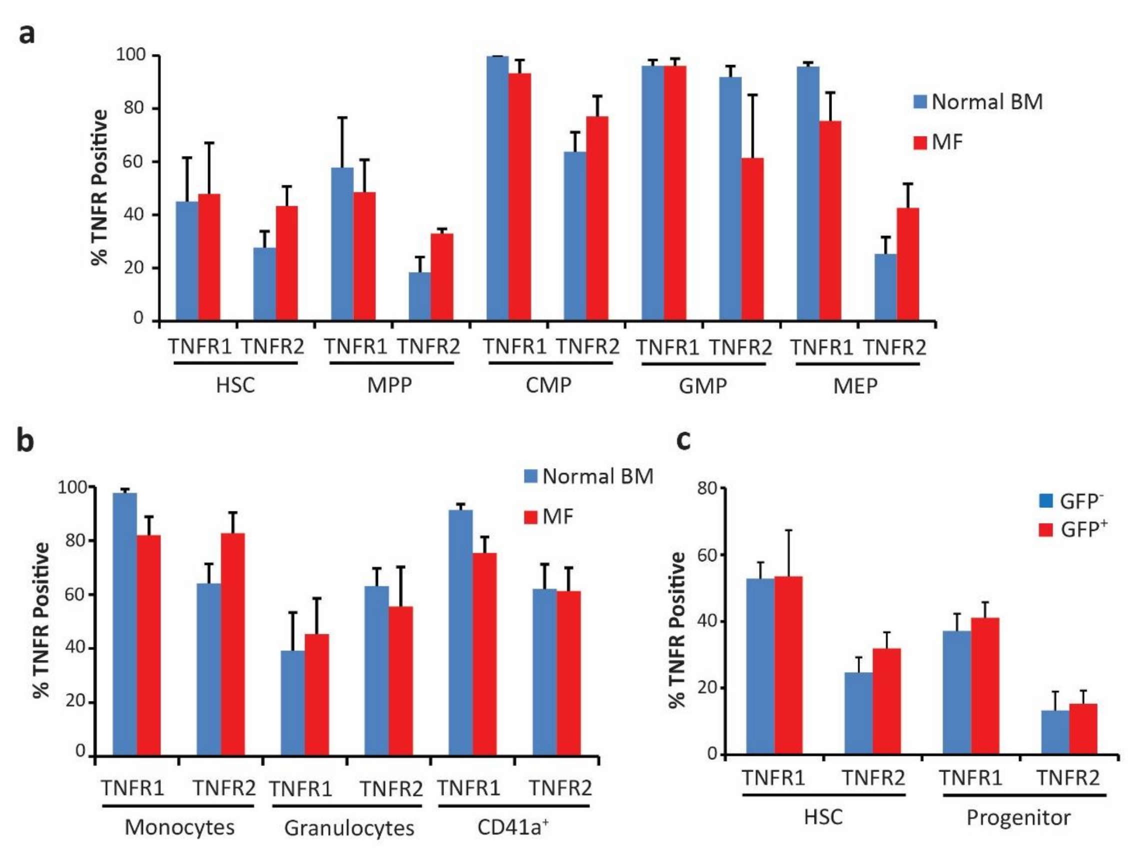

Supplemental Figure 3. TNFR1 and TNFR2 expression is comparable between MF cells and BM controls and in mouse JAK2^{V617F+} compared to JAK2^{V617F-} progenitor cells. (a,b) TNFR1 and TNFR2 receptor expression was measured in (a) primitive and (b) mature hematopoietic cell subsets from MF patient samples (n=3 for primitive and n=5 for mature populations) and normal BM (n=5). There were no significant differences in receptor expression. (c) Assessment of TNFR1 and TNFR2 expression in BM HSCs (Lin$^-$Kit$^+$CD48$^-$CD150$^+$) and bulk progenitor cells (Lin$^-$Kit$^+$CD48$^-$CD150$^-$) from MPN mice also showed no difference between JAK2^{V617F+} and JAK2^{V617F-} cells.

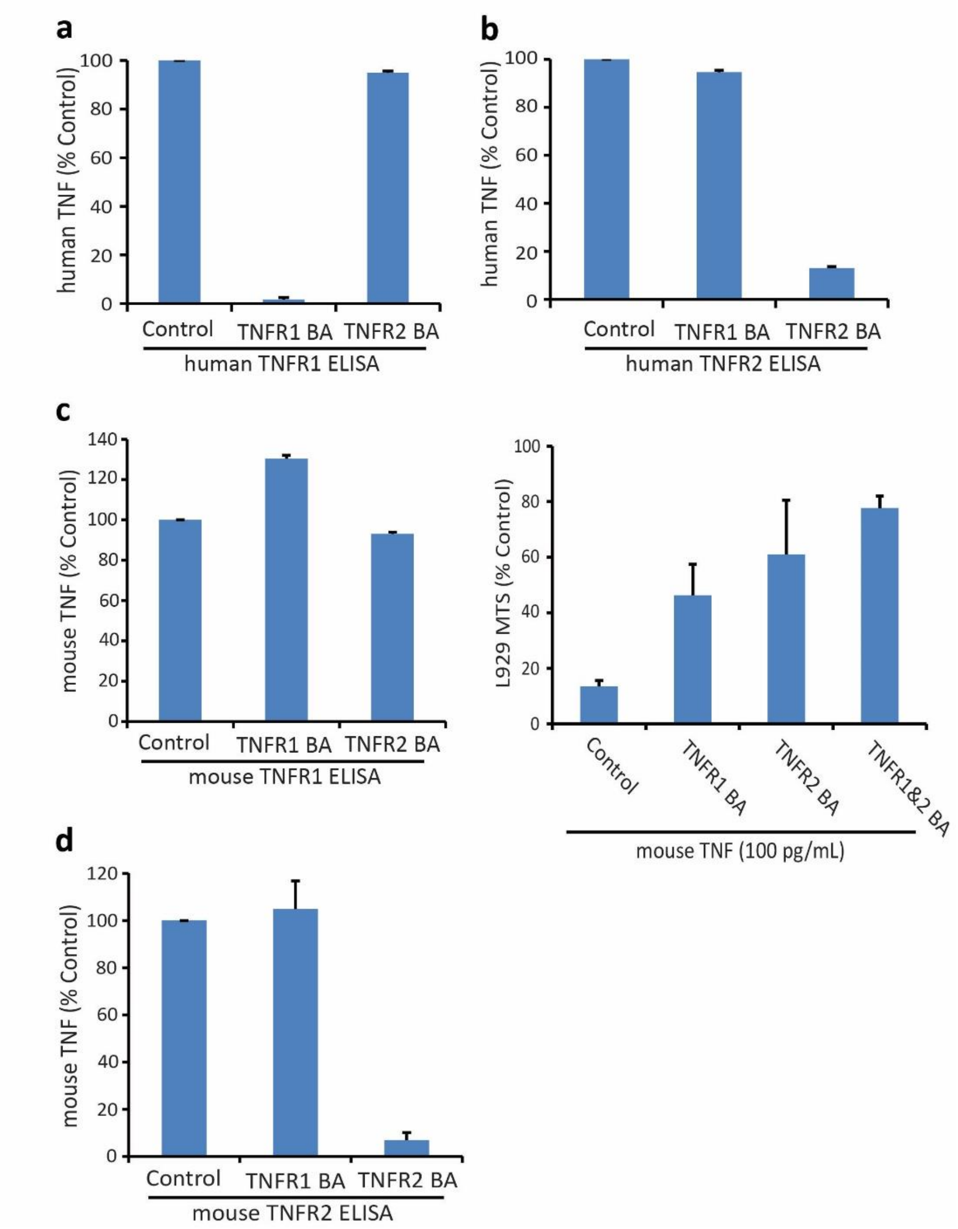

Supplemental Figure 4. TNFR1 and TNFR2 BAs selectively block TNFR1 or TNFR2 respectively. In ELISA assays with human TNFR1 or TNFR2, (A) the TNFR1 BA (10 µg/mL) inhibits TNF (10 ng/mL) binding to plates coated with TNFR1 protein (25ng) but not TNFR2 protein (25ng) and (B) the TNFR2 BA (10 µg/mL) inhibits TNF binding to plates coated with TNFR2 protein (25 ng) but not TNFR1 protein (25 ng). (C) The mouse TNFR1 BA (10 µg/mL) did not inhibit binding of mouse TNF (10 ng/mL) in the ELISA assay but reduced mouse TNF cytotoxicity in the L929 cell assay. (D) The mouse TNFR2 BA (10 µg/mL) inhibited binding of mouse TNF (10 ng/mL) to plates coated with TNFR2 protein (25 ng) but not TNFR1 protein (25 ng).

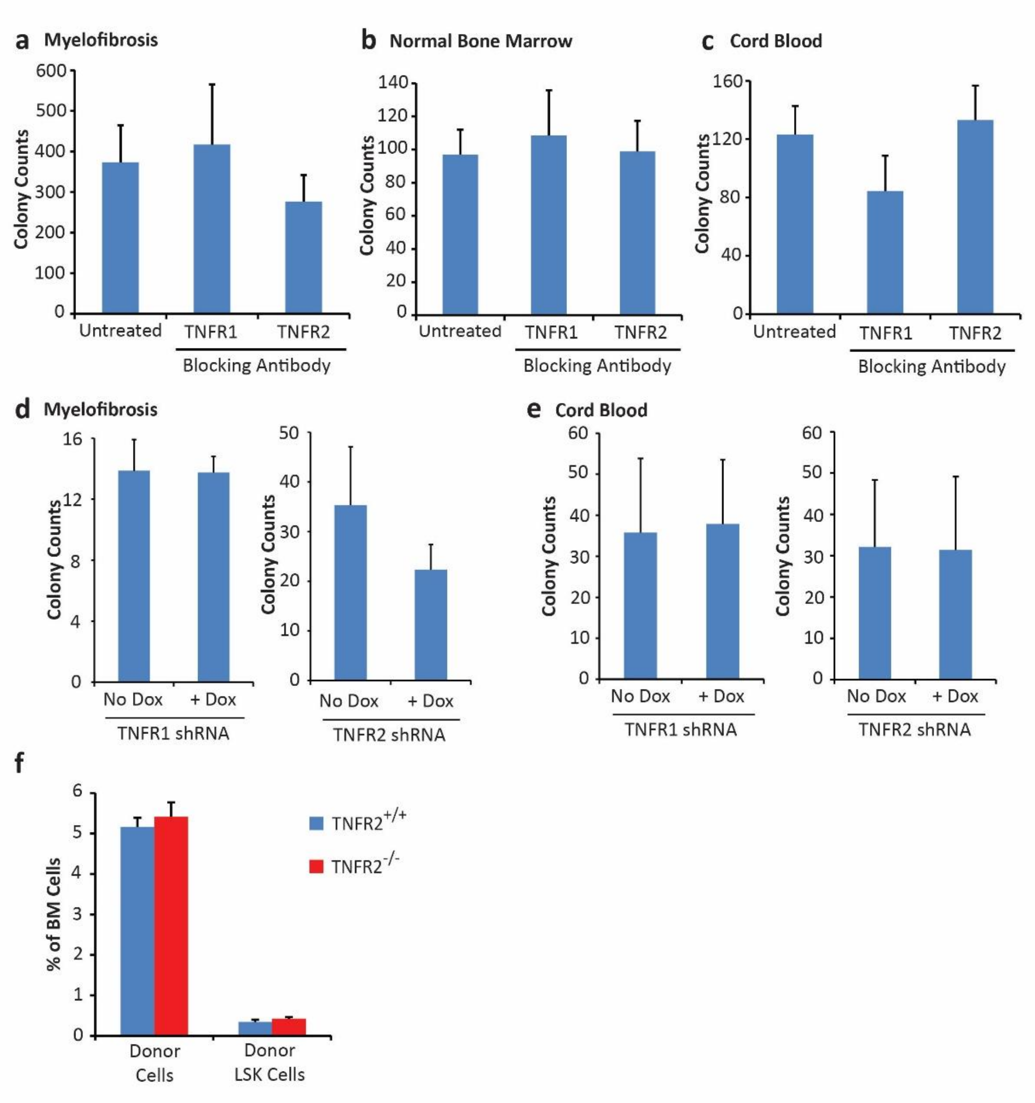

Supplemental Figure 5. Colony numbers from human samples and BM homing of murine donor cells. (a-c) Colony counts from $CD34^+$ cells from (a) myelofibrosis, (b) normal BM or (c) cord blood samples treated with or without TNF receptor blocking antibodies. (d,e) Colony counts from $CD34^+$ cells infected with TNF receptor shRNAs from (d) myelofibrosis samples or (e) human cord blood. Additional details provided in the legend for Figure 2. (f) BM cells from 5-FU-treated $TNFR2^{+/+}$ or $TNFR2^{-/-}$ mice were stained with the lipophilic dye DiO and transplanted into irradiated recipients. After 48 hours, BM cells were harvested from recipient mice and the percent of DiO positive (donor cells) and LSK cells was determined by flow cytometry.

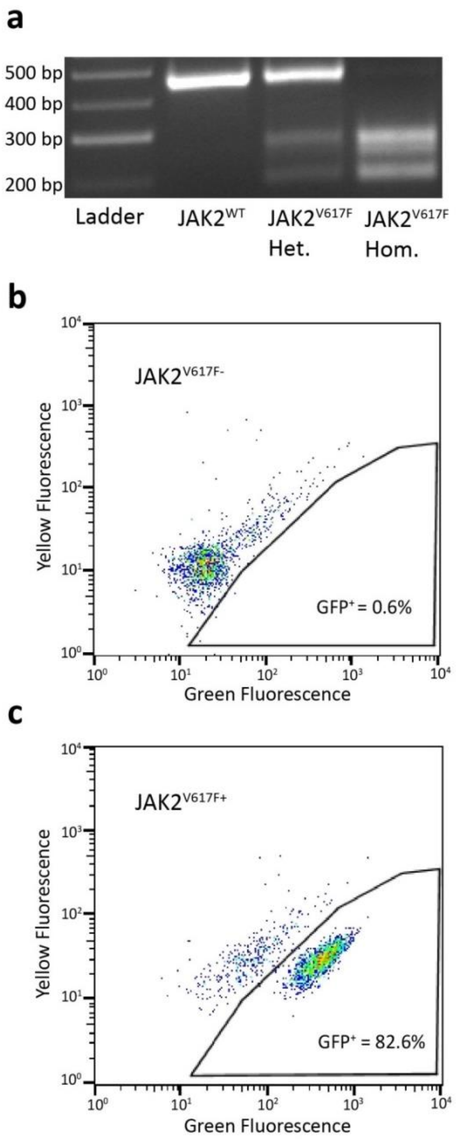

Supplemental Figure 6. Genotyping JAK2 in hematopoietic colonies. (a) In DNA isolated from human colonies, digestion of a JAK2 PCR product with BsaXI identifies JAK2^{WT} colonies with a single band at 460 bp, JAK2^{V617F} heterozygous colonies with 3 bands at 460, 241 and 189 bp, and JAK2^{V617F} homozygous colonies with 2 bands at 241 and 189 bp. (b,c) Mouse colonies are analyzed by flow cytometry where cells from (b) JAK2^{V617F-} colonies lack GFP expression and cells from (c) JAK2^{V617F+} colonies are predominantly GFP^{+}.

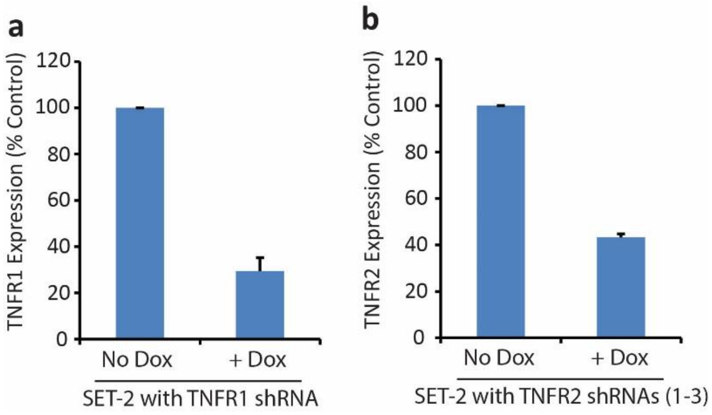

Supplemental Figure 7. TNFR shRNAs reduce mRNA expression of TNFRs in the JAK2^{V617F+} SET-2 cell line. (a) SET-2 cells infected with a doxycycline inducible TNFR1 shRNA, show reduced expression of TNFR1 mRNA by qPCR analysis in the presence of doxycycline (200 ng/mL). (b) TNFR2 mRNA expression was inhibited by a mixture of 3 different doxycycline inducible shRNAs in SET-2 cells when treated with doxycycline (200 ng/mL).

Supplemental Tables

Supplemental Table 1. MF samples

**TNF expression**			
**Mutation**	**Current Treatment**	**Age**	**Sex**
CALR	fedratinib	68	F
JAK2^{V617F}	ruxolitinib	67	F
JAK2^{V617F}	no current therapy	78	F
JAK2^{V617F}	ruxolitinib	73	M
JAK2^{V617F}	ruxolitinib	63	M
**TNF receptor expression**			
**Mutation**	**Current Treatment**	**Age**	**Sex**
JAK2^{V617F}	busulfan	80	F
triple negative	recently stopped pacritinib and darbapoietin	79	F
MPL	pegylated interferon and aranesp	78	M
JAK2^{V617F}	pegylated Interferon	50	M
JAK2^{V617F}	ruxolitinib and 5-azacitidine	77	M
**Clonogenic assay with TNF receptor blocking antibodies**			
**Mutation**	**Current Treatment**	**Age**	**Sex**
JAK2^{V617F}	ruxolitinib	63	M
JAK2^{V617F}	baracitinib	77	F
JAK2^{V617F}	hydroxyurea	62	F
JAK2^{V617F}	ruxolitinib	60	M

Clonogenic assay with TNF receptor shRNAs			
**Mutation**	**Current Treatment**	**Age**	**Sex**
$JAK2^{V617F}$	anegralide	61	M
$JAK2^{V617F}$	ruxolitinib	68	F
$JAK2^{V617F}$	ruxolitinib	76	M
$JAK2^{V617F}$	ruxolitinib	68	F
**XIAP and MAPK8 RNA expression**			
**Mutation**	**Current Treatment**	**Age**	**Sex**
$JAK2^{V617F}$	none	67	F
triple negative	none	80	F
$JAK2^{V617F}$	none	60	M
CALR	ruxolitinib	47	F
JAK2 and MPL negative	recently stopped ruxolitinib	67	M
**cIAP immunofluorescence**			
**Mutation**	**Current Treatment**	**Age**	**Sex**
$JAK2^{V617F}$	none	79	M
$JAK2^{V617F}$	none	70	F
$JAK2^{V617F}$	none	77	F
$JAK2^{V617F}$	none	57	M
**cIAP histology**			
**Mutation**	**Current Treatment**	**Age**	**Sex**
$JAK2^{V617F}$	hydroxyurea	69	M
$JAK2^{V617F}$	pegylated interferon	70	F
$JAK2^{V617F}$	unknown	69	F
$JAK2^{V617F}$	unknown	60	F
CALR	none	68	M
CALR	hydroxyurea and anagrelide	67	M
CALR	hydroxyurea	86	M
CALR	unknown	82	F
**Clonogenic assay with birinapant**			
**Mutation**	**Current Treatment**	**Age**	**Sex**
$JAK2^{V617F}$	none	55	F
$JAK2^{V617F}$	none	68	M
$JAK2^{V617F}$	momelotinib	85	M
**NF-kB reporter assay**			
**Mutation**	**Current Treatment**	**Age**	**Sex**
$JAK2^{V617F}$	ruxolitinib	69	M
$JAK2^{V617F}$	thalidimide and hydroxyurea	82	F
$JAK2^{V617F}$	ruxolitinib and 5-azacitidine	77	M
MPL	ruxolitinib	77	M
**XIAP and MAPK8 ectopic expression**			

Mutation	Current Treatment	Age	Sex
$JAK2^{V617F}$	ruxolitinib	46	M
$JAK2^{V617F}$	ruxolitinib and hydroxyurea	82	F
$JAK2^{V617F}$	ruxolitinib and lenalidomide	76	M
**cIAP immunofluorescence after XIAP ectopic expression**			
**Mutation**	**Current Treatment**	**Age**	**Sex**
$JAK2^{V617F}$	ruxolitinib and hydroxyurea	82	F
$JAK2^{V617F}$	ruxolitinib and lenalidomide	76	M
MPL	ruxolitinib	77	M

Supplemental Table 2. Normal bone marrow samples

**TNF expression**		
**Patient information / sample type**	**Age**	**Sex**
Referred for neutrophilia, BM biopsy was normal	45	F
Seen for leukocytosis, BM biopsy was normal	47	F
Burkitt's lymphoma, no BM involvement	18	M
Idiopathic thrombocytopenic purpura, no BM involvement	27	F
Referred for potential PV, BM biopsy was normal	53	M
Referred for leukocytosis, BM biopsy was normal	60	F
Referred for mastocytosis, BM biopsy was normal	24	F
Diffuse large B-cell lymphoma, no BM involvement	39	M
Diffuse large B-cell lymphoma, no BM involvement	64	F
Hodgkin's lymphoma, no BM involvement	23	M
**TNF receptor expression**		
**Patient information / sample type**	**Age**	**Sex**
Diffuse large B-cell lymphoma, no BM involvement	26	M
Diffuse large B-cell lymphoma, no BM involvement	31	F
Diffuse large B-cell lymphoma, no BM involvement	63	M
Diffuse large B-cell lymphoma, no BM involvement	55	M
Follicular lymphoma, no BM involvement	67	F
Diffuse large B-cell lymphoma, no BM involvement	58	F
**Clonogenic assay with TNF receptor blocking antibodies**		
**Patient information / sample type**	**Age**	**Sex**
Femoral head sample	42	M
Femoral head sample	74	M
Femoral head sample	62	M
**XIAP and MAPK8 RNA expression**		
**Patient information / sample type**	**Age**	**Sex**
Macrocytosis, BM biopsy was normal	55	F
Referred for leukocytosis, BM biopsy was normal	22	F
Diffuse large B-cell lymphoma, no BM involvement	82	M

**cIAP immunofluorescence**		
**Patient information / sample type**	**Age**	**Sex**
Referred for leukocytosis, BM biopsy was normal	54	M
Diffuse large B-cell lymphoma, no BM involvement	82	M
Diffuse large B-cell lymphoma, no BM involvement	77	M
**Clonogenic assay with birinipant**		
**Patient information / sample type**	**Age**	**Sex**
Femoral head sample	55	M
Femoral head sample	68	M
Femoral head sample	85	F
**NF-kB reporter assay**		
**Patient information / sample type**	**Age**	**Sex**
Femoral head sample	47	M
Femoral head sample	88	F
Femoral head sample	68	M

Supplemental Table 3. Human Lineage Markers

Compartment	Markers
HSC	CD34+ CD38- CD90+ CD45RA-
CMP	CD34+ CD38+ CD123+ CD45RA-
GMP	CD34+ CD38+ CD123+ CD45RA+
MEP	CD34+ CD38+ CD123- CD45RA-
CLP	CD34+ CD38+ CD7+ or CD10+
Monocyte	CD45+CD14+CD16-
Granulocyte	CD45+CD14-CD16+
T lymphocyte	CD45+CD3+
B lymphocyte	CD45+CD19+

Supplemental Table 4. Antibodies for Lineage Discrimination in Human Samples

Name	Vendor	Catalog #
CD45RA FITC	BD Biosciences	347723
CD34 PE-Cy7	BD Biosciences	348791
CD38 APC	BD Biosciences	340439
CD45 APC-H7	BD Biosciences	641399
CD7 FITC	BD Biosciences	340737
CD10 PE	BD Biosciences	340921
CD123 PE	BD Biosciences	340545
CD90 PerCP-Cy 5.5	BD Biosciences	561557
CD14 PE	BD Biosciences	562691
CD16 PE-Cy7	BD Biosciences	557744
CD3 FITC	BD Biosciences	555332
CD19 PerCP-Cy5.5	BD Biosciences	561295
CD41a APC	BD Biosciences	559777
TNF-α V450	BD Biosciences	561311

TNFR1 AF700	Novus Biologicals	NBP2-37724AF700
TNFR2 PE	Invitrogen	TNFR7504

Supplemental Table 5. Mouse Lineage Markers

Compartment	Markers
HSC-enriched	Lin- c-kit+ CD48- CD150+
CMP	Lin- c-kit+ CD34+ FcγRII/RIII^{Lo}
GMP	Lin- c-kit+ CD34+ FcγRII/RIII^{Hi}
MEP	Lin- c-kit+ CD34- FcγRII/RIII^{Lo}

Supplemental Table 6. Antibodies for Lineage Discrimination in Murine Samples

Name	Vendor	Catalog #
c-kit (CD117) APC-eFluor780	eBioscience	41-1171-82
CD48 PerCP-eFluor710	eBioscience	46-0481-82
CD150 APC	eBioscience	17-1501-81
FcγRII/RIII (CD16/32) PE-Cy7	eBioscience	25-0161-82
CD34 PE	BD Biosciences	551387
TNFα eFluor450	eBioscience	48-7321-82
Lineage antibody cocktail	BD Biosciences	51-9006957
Lineage Cell Depletion Kit	Miltenyi Biotec	130-090-858
B220 PE-Cy7	eBioscience	25-0452-81
GR1 APC	eBioscience	17-5931-82
CD4 APC-eFluor780	eBioscience	47-0042-82
CD11b PE	eBioscience	12-0112-82
TNFR1 APC	Novus Biologicals	NB110-85469APC
TNFR2 PE	BD Biosciences	550086

Supplemental Table 7. TNF Receptor Blocking Antibodies

Species	Target	Vendor	Catalog #
Human	TNFR1	R&D Systems	MAB225
Human	TNFR2	R&D Systems	MAB726
Mouse	TNFR1	R&D Systems	MAB430
Mouse	TNFR2	R&D Systems	MAB426

Supplemental Table 8. TNF Receptor shRNA Details

Target	Sequence
TNFR1	CCACAGAGCCTAGACACTGAT
TNFR2	CCTGGGAATGCAAGCATGGAT
TNFR2	CCCTTCTCCAAGGAGGAATGT
TNFR2	CACCCTGGAATCAAGATGTCA
Hairpin Insert	ACCGGCCACAGAGCTTAGACATTGATGTTAATATTCATAGCATCAGTGTCTAGGCTCTGTGGTTTT
Vector	pRSIT12-U6Tet-sh-CMV-TetRep-2A-TagRFP-2A-Puro

Supplemental Table 9. CD45 Discrimination Antibodies

Name	Vendor	Catalog #
CD45.1-APC, clone A20	eBioscience	17-0453-81
CD45.2-PE, clone 104	eBioscience	12-0454-82

Supplemental Table 10. qPCR Primers

Species	Target	Forward Primer	Reverse Primer
human	XIAP	GGGGTTCAGTTTCAAGGACA	CGCCTTAGCTGCTCTTCAGT
human	MAPK8	TTGGAACACCATGTCCTGAA	ATGTACGGGTGTTGGAGAGC
human	GUS	GAAAATATGTGGTTGGAGAGCTCATT	CCGAGTGAAGATCCCCTTTTTA
mouse	Xiap	TTGGAACATGGACATCCTCA	TGCCCCTTCTCATCCAATAG
mouse	Mapk8	AGAAACTGTTCCCCGATGTG	TGATGTATGGGTGCTGGAGA
mouse	Gapdh	GGCATTGCTCTCAATGACAA	TGTGAGGGAGATGCTCAGTG

Supplemental Methods

JAK2^{V617F} murine MPN

Murine MPN was induced by retroviral infection of BM cells isolated from 5-fluorouacil (5-FU; APP Pharmaceuticals, Inc., Shaumburg, IL) treated Balb/c (The Jackson Laboratory, Bar Harbor, ME) donor mice with MSCV-IRES-JAK2^{V617F}-GFP as described.[1] Balb/c female recipient mice (6-8 weeks old) were subject to a split dose 900 Gy whole-body irradiation with an RS 2000 X-ray irradiator (Rad Source, Suwanee, GA) and injected via tail vein with 3 x 10^5 transduced cells/mouse BM cells. Sample sizes were chosen based on previous experiments characterizing the disease model.[1] All animal studies were performed under an approved protocol from the University of Utah Institutional Animal Care and Use Committee.

Treatment of mice with MPN with anti-TNF therapeutics

MPN was induced in Balb/c mice as described in the "JAK2^{V617F} murine MPN" methods. In the etanercept (Enbrel®; Amgen, Thousand Oaks, CA) study, mice were treated by subcutaneous injection with normal saline (n=9, control), 25 µg etanercept (n=5) or 50 µg etanercept (n=12) every other day. In a separate experiment we used CNTO5048[2], which is a monoclonal IgG2 antibody specific for mouse TNF, and CNTO6601, the isotype-matched IgG2 negative control (both kindly provided by Janssen Research & Development, Radnor, PA). CNTO5048 and CNTO6601 were both administered at 8 mg/kg by intraperitoneal injection twice a week, with 11 mice in each dose group. Mice were randomized based on initial GFP measurements to ensure that the mean starting values were equal between groups. In both studies, blood counts were analyzed every other week with a HemaTrue® veterinary blood analyzer (Heska, Loveland, CO) following sub-mandibular collection and the proportion of GFP$^+$ was quantified as a percentage of total WBCs with a Guava easyCyte flow cytometer (Millipore, Billerica, MA). The investigators were not blinded to the dose group. Following completion of the study mice were euthanized by exsanguination and subjected to autopsy. Plasma was isolated and used to determine the pharmacodynamic activity of the anti-TNF agents by measuring TNF cytotoxicity in L929 cells.[3] L929 cells were treated with sterilized plasma samples or recombinant mouse TNF (R&D Systems) in the presence of 2 µg/mL actinomycin D (Sigma-Aldrich). Toxicity was determined after 20 hours by addition of an MTS reagent (CellTiter 96®, Promega, Madison, WI) and absorbance at 490 nm was read with an Epoch plate

reader (BioTek, Winooski, Vermont). The L929 cells were confirmed to be mycoplasma free, but had not been authenticated.

Competitive repopulation assay

We used B6.SJL-Ptprca Pepcb/BoyJ mice expressing Ptprc[a] (CD45.1)[4], as TNFR2$^{+/+}$ mice and the TNFR2 deficient strain B6.129S2-Tnfrsf1btm1Mwm/J (TNFR2$^{-/-}$)[5] that express the standard Ptprc[b] (CD45.2). BM cells were harvested from equal numbers of 5-FU treated CD45.1 and TNFR2$^{-/-}$ mice aged 6-8 weeks. Lineage depletion was performed using a Direct Lineage Cell Depletion Kit, (Miltenyi Biotec) with separation on an autoMACS Pro Separator (Miltenyi Biotec), prior to infection with the MSCV-IRES-JAK2^{V617F}-GFP retrovirus. The percentage of GFP$^+$ cells from each mouse strain was measured by FACS and equal numbers of Lin$^-$GFP$^+$ cells from each strain were combined. A total of 5,000 Lin$^-$ (~10% GFP$^+$) cells, from each strain, were transplanted along with 200,000 CD45.1 whole BM cells, by tail vein injection, into 8 CD45.1 female recipient mice (6-8 weeks old) which had been subjected to 11.5 Gy whole-body irradiation. To monitor disease, flow cytometry of peripheral blood cells following sub-mandibular collection was performed and the proportion of GFP$^+$ cells in CD45.1$^+$ and CD45.2$^+$ fractions was determined.

Bone marrow homing

TNFR2$^{+/+}$ or TNFR2$^{-/-}$ (see description above) donor mice were treated with 5-FU 5 days prior to harvest. BM cells from donor mice were stained with the carbocyanine dye DiO (5 µM; Molecular Probes, Eugene OR) for 20 minutes at 37 °C. TNFR2$^{+/+}$ recipient mice were subject to a split dose, 11.5 Gy whole-body irradiation. Donor cells (500,000 per mouse) were transplanted by tail vein injection into 3 recipient mice for each donor cell type. After 48 hours, BM cells were isolated from recipient mice and analyzed for DiO expression by flow cytometry. In addition, markers for Sca-1, c-Kit and mature lineages were included for the identification of Lin$^-$, Sca-1$^+$, c-Kit$^+$ (LSK) cells.

JAK2^{V617F} genotyping

Colonies from human CD34$^+$ cells were genotyped by isolating DNA from individual colonies, amplifying JAK2 by nested PCR and digesting with BsaXI as described.[6] DNA was isolated with an Allprep DNA/RNA Microkit (Qiagen, Hilden, Germany). The first PCR step was performed with forward primer gtcaagcctgtttgactggcattattc and reverse primer caatgttatgttgaactgccataatc. The second PCR step was performed with 1.5 µL of DNA template from step 1 with forward primer gggtttcctcagaacgttga and reverse primer tcattgctttcctttttcacaa, which amplified a 460-bp reaction product. The product from step 2 was digested with 0.75 U of BsaXI (New England Biolabs, Ipswich, MA) overnight at 37 °C and the product was run on a 2% agarose gel. Since the JAK2^{V617F} mutation removes the BsaXI restriction site, the homozygous JAK2^{V617F} mutants were identified by a single 460-bp band, JAK2^{WT} colonies displayed a 241-bp and a 189-bp band, while the JAK2^{V617F} heterozygous mutants displayed all 3 bands (Supplemental Figure 3A). Genotyping for JAK2^{V617F} in mouse BM derived colonies was performed by plucking single colonies, washing with PBS to remove methylcellulose and analyzing GFP expression on a Guava easyCyte flow cytometer (EMD Millipore, Darmstadt, Germany). Colonies were scored as JAK2^{V617F+} when >50% of the cells from a colony were positive for GFP expression, although the majority were <1% or >80% GFP$^+$ (Supplemental Figure 3B, C).

Immunofluorescence for cIAP

Cells were washed in PBS and spun onto positively charged glass slides (VWR, Radnor, PA). Cells were fixed on the slide by incubation with 4% paraformaldehyde (Electron Microscopy Sciences, Hatfield, PA) for 20 minutes at room temperature. Cells were then blocked and permeabilized by addition of PBS with 5% bovine serum albumin fraction V (BSA, Thermo Fisher Scientific) and 0.1% Triton X-100 (Sigma-Aldrich) for 15 minutes at room temperature. The cIAP1/2 rabbit IgG polyclonal antibody (Thermo Fisher Scientific; Catalog # PA5-20066) was diluted in PBS/5%BSA/0.1% Triton X-100 to a final concentration of 10 µg/mL and incubated with cells at 4°C for 16 hours. Slides were washed 5 times with PBS and then Alexa Fluor 488 or 594 goat anti-rabbit secondary antibody was added at 4 µg/mL and incubated for 90 minutes at room temperature. Slides were washed 5 times with PBS and then 4′, 6-diamidino-2-phenylindole (DAPI, Sigma-Aldrich) was added to the slides at 2 µg/mL in PBS and incubated for 5 minutes at room temperature. Slides were washed 3 times with PBS and then a No. 1.5 cover slip was mounted to each slide with a drop of ProLong Gold antifade reagent (Thermo Fisher Scientific). Slides were imaged with an Axioskop 2 (Carl Zeiss Microscopy, Jena, Germany) with a 40X objective or a Leica TCS SP8 X (Leica Microsystems, Wetzlar, Germeny) with a 63X objective. Quantification was performed by manually delineating cytoplasmic regions and measuring the pixel intensity with Adobe Photoshop (Adobe Systems Inc., San Jose, CA) or with Imaris image analysis software (Bitplane, Zurich, Switzerland).

Histology of human bone marrow biopsies

Bone marrow specimens were fixed overnight with AZF (Acetic Zinc Formalin) and decalcified for 6 hours with Gooding and Stewart's decalcification fluid. Four-µm sections were cut with a Leica rotary microtome and air dried on plus glass slides at room temperature. Slides were melted in the oven at 60 °C for 30 minutes to melt the paraffin. Staining procedures were performed with a Ventana BenchMark ULTRA (Roche, Basel, Switzerland) automated staining system. Slides were de-paraffinized with EZ Prep solution (Roche) and pretreated with CC1 cell conditioning solution (Roche) for 36 minutes for antigen retrieval. The cIAP1/2 polyclonal antibody (Catalog # PA5-20066, ThermoFisher Scientific) was applied at 1.7 µg/mL for 32 minutes at 37 °C. Detection was performed with the ultraView Universal DAB Detection Kit (Roche). Hematoxylin counterstain was then applied for 8 minutes. Slides were removed from the autostainer and placed in a dH_2O/DAWN mixture (1 mL of DAWN, 500 mL of H_2O) to remove residual oil from the autostainer. Slides were dehydrated in graded alcohols (70%, 95% and 100%) and dipped 10 times each in 4 changes of xylene and cover slipped.

qPCR expression analysis

RNA was isolated from CD34$^+$ cell pellets (5 x 10^4 – 2 x 10^5 cells/pellet) with an RNeasy Micro Kit (Qiagen) including an on-column DNA digestion step according to the manufactures instructions. For each sample cDNA conversion was performed with iScript Reverse Transcription Supermix (Bio-Rad, Hercules, CA). Expression data was generated by performing qPCR with target specific primers (Supplemental Table of qPCR primers) and SsoFast EvaGreen Supermix (Bio-Rad) on a C1000 Thermal Cycler (Bio-Rad). Expression was normalized by identifying the Ct value for each condition and normalizing the result of test genes with the housekeeping gene GUS by calculating the ΔCt (GUS Ct – test gene Ct). Normalized expression is then calculated as $2^{\Delta Ct}$.

Microarray data analysis

CEL files from the output of Affymetrix GeneChip Command Console were used as input for determining differential gene expression among the various treatments. One of the samples (Untreated-GFP$^+$

replicate #3) was excluded because it failed QC parameters according to the Bioconductor affyQCReport package.[7] The data were normalized using the GCRMA[8] linear models and empirical Bayesian methods for assessing differential expression in microarray experiments and fold-change in expression was assessed relative to the Untreated-GFP$^-$ group.

NF-κB Reporter Assay

MF CD34$^+$ cells were infected with lentiviral particles containing the Cignal Lenti NF-κB Reporter (Qiagen), that has tandem repeats of an NF-κB transcriptional response element, or a negative control reporter. Cells were incubated with virus for 16 hours and then resuspended in fresh medium. At 72 hours post infection, cells were transferred to 96-well white plates (ThermoFisher Scientific). Cells were treated with TNFR blocking antibodies at 10 µg/mL for 30 minutes prior to stimulation with TNF (1 ng/mL). Addition of ONE-Glo luciferase substrate (50 µL, Promega) was performed at the indicated timepoints after stimulation and luminescence was read on a GloMax-Multi Detection System (Promega).

References (Supplemental Methods)

1. Bumm TG, Elsea C, Corbin AS, Loriaux M, Sherbenou D, Wood L*, et al.* Characterization of murine JAK2V617F-positive myeloproliferative disease. *Cancer research* 2006 Dec 1; **66**(23)**:** 11156-11165.

2. Lei B, Dawson HN, Roulhac-Wilson B, Wang H, Laskowitz DT, James ML. Tumor necrosis factor alpha antagonism improves neurological recovery in murine intracerebral hemorrhage. *Journal of Neuroinflammation* 2013; **10**(1)**:** 103.

3. Shiau MY, Chiou HL, Lee YL, Kuo TM, Chang YH. Establishment of a consistent L929 bioassay system for TNF-alpha quantitation to evaluate the effect of lipopolysaccharide, phytomitogens and cytodifferentiation agents on cytotoxicity of TNF-alpha secreted by adherent human mononuclear cells. *Mediators of Inflammation* 2001; **10**(4)**:** 199-208.

4. Janowska-Wieczorek A, Majka M, Kijowski J, Baj-Krzyworzeka M, Reca R, Turner AR*, et al.* Platelet-derived microparticles bind to hematopoietic stem/progenitor cells and enhance their engraftment. *Blood* 2001; **98**(10)**:** 3143-3149.

5. Erickson SL, de Sauvage FJ, Kikly K, Carver-Moore K, Pitts-Meek S, Gillett N*, et al.* Decreased sensitivity to tumour-necrosis factor but normal T-cell development in TNF receptor-2-deficient mice. *Nature* 1994 Dec 08; **372**(6506)**:** 560-563.

6. Steensma DP. JAK2 V617F in myeloid disorders: molecular diagnostic techniques and their clinical utility: a paper from the 2005 William Beaumont Hospital Symposium on Molecular Pathology. *The Journal of molecular diagnostics : JMD* 2006 Sep; **8**(4)**:** 397-411; quiz 526.

7. Huber W, Carey VJ, Gentleman R, Anders S, Carlson M, Carvalho BS*, et al.* Orchestrating high-throughput genomic analysis with Bioconductor. *Nature methods* 2015 Feb; **12**(2)**:** 115-121.

8. Wu Z, Irizarry RA, Gentleman R, Martinez-Murillo F, Spencer F. A Model-Based Background Adjustment for Oligonucleotide Expression Arrays. *Journal of the American Statistical Association* 2004; **99**(468)**:** 909-917.
